# Association of food neophobia and food disgust with the willingness, benefits, and risks of insect food consumption among Chinese university students

**DOI:** 10.3389/fnut.2025.1613932

**Published:** 2025-07-14

**Authors:** Hua Tian, Jie Chen

**Affiliations:** ^1^College of Life Science, Xinyang Normal University, Xinyang, China; ^2^School of Marxism, Xinyang Normal University, Xinyang, China

**Keywords:** food neophobia, food disgust, insect food, familiarity, willingness

## Abstract

**Introduction:**

Edible insects with high protein content are sufficient to meet the growing global demand for protein. However, some individuals have negative psychological reactions such as phobia and disgust toward insect foods; therefore, the large-scale promotion of insect foods has progressed slowly. This study investigated the impact of food neophobia and food disgust on the willingness, benefits, and risks of insect food consumption among Chinese university students.

**Methods:**

In 2023, 560 university students aged 18–25 years were recruited to fill an online questionnaire. The data were analyzed using a Mann–Whitney U test.

**Results:**

Most university students were unfamiliar and unwilling to consume insect foods, with higher levels of food neophobia and disgust indicating greater unwillingness to consume insect foods. Regardless of food neophobia and disgust levels, both groups agreed on the benefits of consuming insect foods and believed that consuming insect foods carried high risks.

**Conclusion:**

These findings underscored the significance of promoting and enhancing positive cognitions surrounding insect foods, and eliminating negative stereotypes about insect foods, especially those university students with high food neophobia or disgust. In addition, the findings provide reference for studying the psychological mechanism of insect food consumption and guiding young people to consume insect foods.

## 1 Introduction

As global population grows and resources diminish, humans face the double pressure of biological resource shortage and environmental pollution. Therefore, seeking new alternative protein resources is necessary for sustainable development (1). Edible insects have high protein and nutritional value, which can help solve the problems of global malnutrition and food insecurity (2). Insect consumption is a traditional practice in several countries (3). Apart from Europe and North America, approximately 1,500–2,000 species of insects and other invertebrates are consumed by 3,000 ethnic groups across 113 countries in Asia, Australia, and Central and South America (4).

However, in Western societies where a culture of insect consumption does not exist, there is a traditional culture of feeling disgusts over the idea of consuming insects (5). Disgust is a negative emotion that belongs to the category of basic emotions and is often cited as a primary psychological factor in the rejection of insect-based foods. People in Western societies mistakenly classify insects as a pathogen risk and food pollutants, which is the main reason they generally feel aversion toward foods containing insects (6). Such disgust may also be due to insect foods’ peculiar appearance, especially the sharp or rough shells, barbed needles, wings, and spines of protozoan insect foods. Some insects are soft and slippery, such as a puddle of mush, and the idea of eating them may cause discomfort.

Disgust toward insect foods may also be associated with food neophobia (FN) (7). FN has not yet been classified as a disease but represents a persistent irrational fear of insects and the desire to avoid or run away from them (8, 9). The tendency toward FN is usually a personal choice, but may also manifest as a result of cultural influences, lack of exposure to particular foods, or inherited beliefs (10). Studies have confirmed that consumer familiarity is an important and determining factor in shaping positive attitudes toward edible insect consumption (11, 12), which makes it easier for consumers to accept insect food (13, 14). For example, research on insect consumption among communities in western Kenya has shown that young people, especially those in urban markets, have little knowledge of edible insect species, and therefore find it more difficult to accept insects as a source of food (15).

However, European culture often views insects as dirty, disgusting, dangerous or vectors of disease (16). Such negative attitudes may lead to a greater fear of new insect foods, which may negatively affect consumers’ perceptions and willingness to eat food, even if the insect food is a healthy and sustainable food choice (17). FN for insect foods limits dietary diversity and negatively affects the acceptance of new and unfamiliar foods (18). FN, perceived aversion or disgust, concerns about safety risks, and poor sensory quality are the main reasons for negative psychological reactions of fear or aversion to insect foods (19).

The tradition of eating insects in China can be traced back over 3,000 years. Insects are a source of protein and are generally considered healthier and more sustainable alternatives to meat. Eating insect foods is a potential solution to address malnutrition and food insecurity in China (20). Compared with Germans, Chinese people have a more favorable attitude toward and are more willing to eat insect food. FN has a significant impact on the willingness of Chinese and German individuals to eat insect foods (21). Disgust and phobia pose obstacles to spreading the practice of insects consumptions, but stimulating positive emotions can help overcome these psychological barriers (22).

Evaluating young people’s acceptance of new foods such as insect foods is especially important. Driven by curiosity and low risk, they may become the target audience for new food consumption (23). Spanish university students recognize the high nutritional value of insects, particularly their protein content. Advertising and insect knowledge are important factors that influence insect food consumption (24). Polish university students have a low willingness to use edible insects as meat substitutes (25), and the health and quality related characteristics of insect foods are the main factors influencing their willingness to purchase them. College students without FN have a positive attitude toward purchasing foods containing edible insects as ingredients (26). Furthermore, French students are more willing to eat insects than Irish students; however, FN and food aversion or food disgust (FD) are their main obstacles to eating insects (27). Few studies have examined the psychological mechanisms of the acceptance and rejection of insects, especially the attitudes and behaviors of university students in China toward insect foods.

These findings can fill the existing literature gap in Chinese college students’ familiarity with and willingness to consume insect food, and provide initial insights for a deeper understanding of students’ cognitions about, acceptance of, and motivations for consuming insect food. The results are expected to contribute to the psychological mechanism of insect food consumption and the healthy development of this industry, providing theoretical basis for formulating corresponding policies and regulations.

## 2 Materials and methods

### 2.1 Study design

This non-experimental study used quantitative research methods. Online questionnaires were used to collect data exploring the impact of FN and FD on university students’ willingness to consume insect foods and perceptions of benefits and risks of insect food consumption. The online questionnaires were created using Wenjuanxing, which is an online survey platform that provides various data collection and analysis functions. The study was conducted from April to July 2023.

### 2.2 Participants

Individuals participated voluntary (*N* = 572) were anonymously recruited through WeChat groups of Xinyang Normal University to complete online questionnaires. Voluntary participants only needed to click on the questionnaire link or QR code on their smartphones or computers to participate freely and independently. All participants were native Mandarin speakers and students of Xinyang Normal University. If they did not complete the questionnaire (e.g., incomplete answers to questions, missing demographic information, answering some questions instead of all, or responding with the same option for all questions), they were excluded from the study. Finally, 560 participants aged 18–25 years were selected (response rate: 97.90%). The participants were predominantly female (68.8%), with an average age of 19.25 ± 1.19 years (Table 1).

**TABLE 1:** Participant characteristics (*N* = 560).

Items	Frequency	Proportion (%)
**Gender**		
Male	175	31.3
Female	385	68.8
**Age**		
18–25 years old	19.25 ± 1.19	
**Major**		
Humanities	216	38.57
Science and engineering	344	61.43
**Place of residence**		
City	211	37.68
Rural	349	62.32

### 2.3 Data collection

The data were collected through an online questionnaire created using Wenjuanxing. All participants completed the questionnaire, which included the FN scale (Supplementary Table 1), FD scale (Supplementary Table 2), and items for the willingness to consume insect foods, benefits and risks of insect food consumption, and demographic information (Supplementary Table 3). The following three items were also included: (1) Are you familiar with insect foods? (2) Please choose which insect foods you frequently consume. (3) Please list the possible reasons for not eating insect foods (Supplementary Table 4).

### 2.4 Data analysis

Data were analyzed using SPSS V20 (IBM, Armonk, NY, United States), and significant differences between the variables were identified using the Mann–Whitney U test. To study the impacts of FN and FD on the willingness, benefits, and risks of insect food consumption, the averages of 10 FN items and 8 FD items were used separately as cutoff scores. Scores ≥ 31 represented the high FN group, and scores < 31 represented the low FN group. Similarly, scores ≥ 30 represented the high FD group, and scores < 30 represented the low FD group. The overall Kaiser–Meyer–Olkin (KMO) value was 0.910, and the Cronbach’s alpha was 0.873, indicating the excellent reliability and validity of the questionnaire.

### 2.5 Ethics approval

This study was conducted in compliance with the principles of the Declaration of Helsinki. All procedures relevant to the study participants were approved by the Xinyang Normal University Ethics Committee (Approval Number: XFEC-2023-025).

### 2.6 Informed consent

Participation in the study was voluntary. All participants were informed of the study objective and context, and they provided written informed consent for privacy and information management policies.

## 3 Results

### 3.1 University students’ familiarity with and consumption of insect foods

**FIGURE 1:**
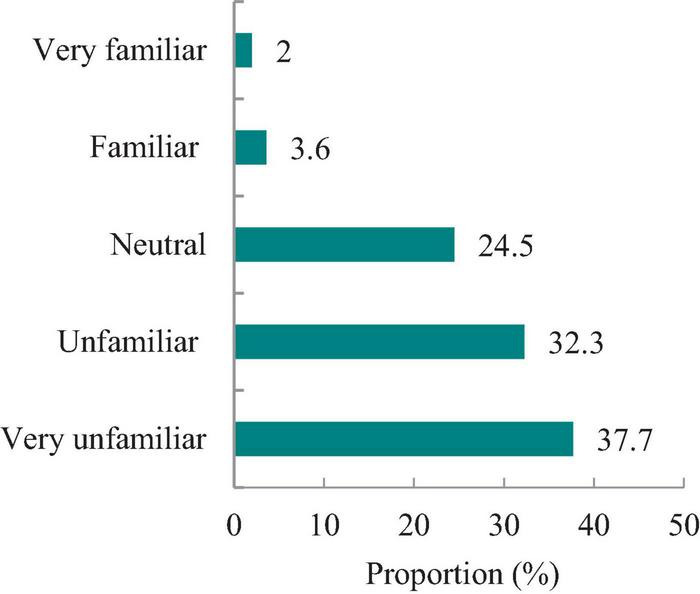
Familiarity with insect foods.

**FIGURE 2:**
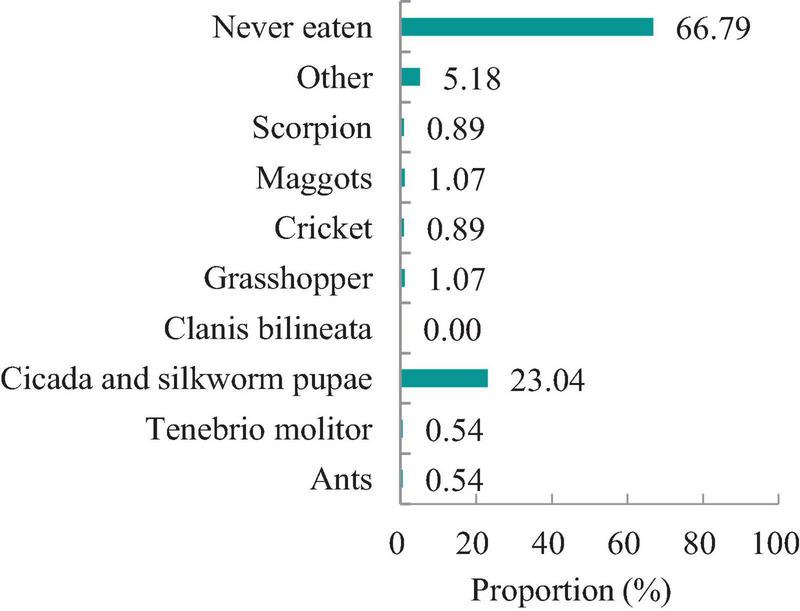
The most eaten insect foods.

**FIGURE 3:**
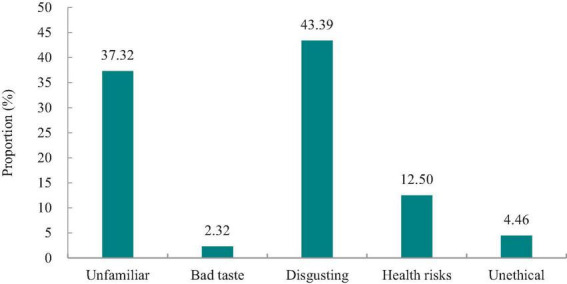
Possible reasons for not liking to eat insect foods.

As shown in Figure 1, 37.7% of university students were very unfamiliar with insect foods, and 32.3% were unfamiliar with insect foods. Only 5.6% of the participants were familiar with insect food. Figure 2 shows the insect foods eaten most commonly by Chinese university students. Approximately 23.04% (*n* = 129) of the participants reported that cicada and silkworm pupae were the most commonly eaten insect foods. Additionally, 66.79% (*n* = 374) reported that they had never consumed insect food, which was a relatively high proportion. Unfamiliarity with and disgust toward insect food were the two main reasons for not consuming insect food (Figure 3).

Tables 2, 3 present participants’ levels of FN and FD. Table 4 showed the results of the in-depth analysis, revealing that the FN (30.50 ± 3.780) of participants who were unfamiliar with insect food was lower than the average (31.03 ± 4.286); however, the FD (31.35 ± 5.794) was higher than the average (30.80 ± 7.258). Furthermore, the FN (31.87 ± 4.836) and FD (31.06 ± 7.716) of participants who were disgusted with insect food were higher than the averages (31.03 ± 4.286 and 30.80 ± 7.258, respectively).

**TABLE 2:** Food neophobia scale (FNS).

	Items	Mean	SD
FNS 1	I am constantly sampling new and different foods (R)	3.15	0.939
FNS 2	I don’t trust new foods	3.46	0.902
FNS 3	If I don’t know what a food is, I won’t try it	3.60	1.015
FNS 4	I like foods from different cultures (R)	2.78	0.929
FNS 5	Ethnic food looks weird to eat	2.79	0.885
FNS 6	At dinner parties, I will try new foods (R)	2.74	0.931
FNS 7	I am afraid to eat things I have never had before	3.00	0.919
FNS 8	I am very particular about the foods I eat	3.13	0.929
FNS 9	I will eat almost anything (R)	3.56	0.983
FNS 10	I like to try ethnic restaurants (R)	2.79	0.84
Total		31.03 ± 4.286	
Cronbach’s alpha		0.749	
Kaiser–Meyer–Olkin		0.727	

5-point Likert scale: ∙ Ranging from 1 for “strongly disagree” to 5 for “strongly agree.” ∙ Each item score varies from 1 to 5. ∙ The total score varies from 10 to 50. ∙ A higher score indicates a higher FN level. “R” stands for “reverse item.”

**TABLE 3:** FD scale.

	Items	Mean	SD
FDS 1	Uncooked animal bones in the mouth	4.03	1.077
FDS 2	Eating with dirty dishes in a restaurant	4.12	1.084
FDS 3	Food donated by an unfamiliar neighbor	3.31	0.962
FDS 4	Eating hard cheese with mold removed	3.80	1.077
FDS 5	Eating apple slices that turned brown when exposed to air	3.54	1.025
FDS 6	Eating unwashed live fish	4.14	1.078
FDS 7	Eating discolored avocado flesh	3.79	1.053
FDS 8	Eating a salad with a snail	4.08	1.082
Total		30.80 ± 7.258	
Cronbach’s alpha		0.949	
Kaiser–Meyer–Olkin		0.928	

5-point Likert scale: ∙ Ranging from 1 for “very not disgusted” to 5 for “strongly disgusted.” ∙ Each item score varies from 1 to 5. ∙ The total score varies from 8 to 40. ∙ A higher score indicates a higher food disgust level.

**TABLE 4:** Specific analysis of the main reasons for not eating insects.

Items	Unfamiliar	Disgusted	*Z*	*P*
	(*n* = 209)	(*n* = 243)		
FN	30.50 ± 3.780	31.87 ± 4.836	–2.863	0.004
FD	31.35 ± 5.794	31.06 ± 7.716	–0.652	0.514

The FN and FD averages (*N* = 560) were 31.03 ± 4.286 and 30.80 ± 7.258, respectively.

In short, most university students were unfamiliar with and unwilling to consume insect foods. The groups of university students who were unfamiliar with or felt disgusted by insect foods had a higher FD than the average. Particularly, the FN and FD of university students who were disgusted by insect food were higher than the average.

### 3.2 Impact of FN on willingness, benefits, and risks of insect food consumption

As shown in Table 5, university students had a lower willingness to consume insect foods, especially the high FN group. University students in the high FN group were significantly more unwilling to consume insect foods at any time (*P* < 0.001). However, when in danger and having to rely on insect food for survival, both the high and low FN groups were willing to consume insect food, with no significant difference between the two groups (*P* = 0.192). The low FN group was more likely to be persuaded to consume insect food or voluntarily do so.

**TABLE 5:** Impact of FN on willingness, benefits, and risks of insect food consumption.

Insect food consumption	Low FN	High FN	*Z*	*P*
**Willingness**				
I won’t eat at any time.	2.84 ± 1.261	3.40 ± 1.40	4.771	< 0.001
I will only eat if I have to rely on it for survival.	3.29 ± 1.126	3.41 ± 1.195	1.303	0.192
I’m not sure if I will consume it.	3.01 ± 1.259	2.78 ± 1.440	−1.774	0.076
Can be persuaded to consume.	2.48 ± 1.097	1.99 ± 1.030	−5.289	< 0.001
I am happy to consume.	2.09 ± 1.014	1.63 ± 0.835	−5.409	< 0.001
**Benefits**				
Nutrition: high protein content, low fat content.	3.52 ± 1.015	3.49 ± 1.152	0.576	0.565
Rich or available: abundant sources.	3.46 ± 1.028	3.24 ± 1.162	−1.718	0.086
Cost: breeding costs are lower than raising cattle.	3.38 ± 1.035	3.29 ± 1.128	−0.263	0.792
Environmental protection: reduced the impact of the commercial meat industry on the environment.	3.31 ± 1.049	3.12 ± 1.191	−1.341	0.180
Cooking: increased consumer choices.	3.23 ± 1.027	2.97 ± 1.075	−2.432	0.015
Health: possible health benefits.	3.31 ± 0.992	3.02 ± 1.065	−2.907	0.004
**Risks**				
Microorganisms or diseases: may be infected with diseases or transmitted by insects.	3.58 ± 0.985	4.11 ± 0.843	6.621	< 0.001
Food fear: insects are disgusting.	3.61 ± 1.031	4.22 ± 0.875	7.043	< 0.001
Poison or toxin: may cause food poisoning.	3.53 ± 0.973	4.05 ± 0.881	6.306	< 0.001
Sensory dislike: taste may not be acceptable or texture may not be good.	3.56 ± 1.041	4.25 ± 0.837	8.050	< 0.001
Insecticides or chemicals: residual chemicals and insecticides on the body.	3.56 ± 1.013	4.14 ± 0.812	6.912	< 0.001
Allergy: may cause body allergies.	3.59 ± 0.978	4.12 ± 0.828	6.556	< 0.001
General health risks: can affect our health.	3.40 ± 0.960	3.92 ± 0.939	6.121	< 0.001
Cronbach’s alpha	0.877			
Kaiser–Meyer–Olkin	0.913			

The FN average (*N* = 560) was 31.03 ± 4.286. The average of the FN items was used as a cutoff score for the low and high groups.

For the benefits of consuming insect food, no significant difference existed between the high and low FN groups in the five dimensions of insect food nutrition (*P* = 0.565), abundant sources (*P* = 0.086), cost (*P* = 0.792), environmental protection (*P* = 0.180), and no benefits (*P* = 0.622). However, significant differences were observed between the two groups for the cooking (*P* = 0.015) and health (*P* = 0.004) dimensions. The groups also differed significantly in the eight risk dimensions associated with the consumption of insect foods (*P* < 0.001). The higher FN indicated a higher perceived risk of consuming insect foods.

Overall, university students in the high FN group were less willing to consume insect-based foods than those in the low FN group. Both groups agreed on the benefits of insect foods but believed that consuming insect foods had higher risks.

### 3.3 Impact of FD on willingness, benefits, and risks of insect food consumption

University students’ FD affected their willingness to consume insect food and perceptions of the benefits and risks of insect food consumption (Table 6). University students in the high FD group were more unwilling to consume insect foods at any time than those in the low FD group, unless their survival was threatened. In both cases, significant differences were observed between the two groups. When university students have low levels of FD, they may voluntarily try or be persuaded by others to consume insect-based food. However, when in danger and having to rely on insect food for survival, both groups were willing to consume insect food, with a significant difference between the two (*P* < 0.001).

**TABLE 6:** Impact of FD on willingness, benefits, and risks of insect food consumption.

Items	Low FD	High FD	*Z*	*P*
**Willingness**				
I won’t eat at any time.	2.85 ± 1.224	3.17 ± 1.398	2.731	0.006
I will only eat if I have to rely on it for survival.	3.09 ± 1.135	3.50 ± 1.136	4.325	< 0.001
I’m not sure if I will consume it.	2.89 ± 1.179	2.95 ± 1.423	0.851	0.395
Can be persuaded to consume.	2.52 ± 1.080	2.16 ± 1.089	−3.931	< 0.001
I am happy to consume.	2.20 ± 0.993	1.74 ± 0.925	−5.772	< 0.001
**Benefits**				
Nutrition: high protein content, low fat content.	3.31 ± 1.092	3.64 ± 1.027	4.225	< 0.001
Rich or available: abundant sources.	3.24 ± 1.073	3.47 ± 1.079	3.151	0.002
Cost: breeding costs are lower than raising cattle.	3.16 ± 1.063	3.47 ± 1.056	4.005	< 0.001
Environmental protection: reduced the impact of the commercial meat industry on the environment.	3.12 ± 1.058	3.32 ± 1.129	2.618	0.009
Cooking: increased consumer choices.	3.00 ± 1.024	3.23 ± 1.059	2.953	0.003
Health: possible health benefits.	3.12 ± 0.977	3.27 ± 1.056	2.200	0.028
**Risks**				
Microorganisms or diseases: may be infected with diseases or transmitted by insects.	3.30 ± 1.045	4.07 ± 0.779	9.327	< 0.001
Food fear: insects are disgusting.	3.33 ± 1.061	4.17 ± 0.837	9.637	< 0.001
Poison or toxin: may cause food poisoning.	3.24 ± 0.991	4.03 ± 0.821	9.737	< 0.001
Sensory dislike: taste may not be acceptable or texture may not be good.	3.29 ± 1.059	4.15 ± 0.847	9.836	< 0.001
Insecticides or chemicals: residual chemicals and insecticides on the body.	3.26 ± 1.028	4.10 ± 0.797	10.078	< 0.001
Allergy: may cause body allergies.	3.29 ± 0.985	4.10 ± 0.797	9.914	< 0.001
General health risks: can affect our health.	3.17 ± 0.977	3.87 ± 0.883	8.268	< 0.001
Cronbach’s alpha	0.877			
Kaiser–Meyer–Olkin	0.913			

The FD average (*N* = 560) was 30.80 ± 7.258. The average of the eight FD items was used as the cutoff scores for the low and high groups.

Regarding the benefits of consuming insect foods, the high FD group believed that the nutrition, sources, cost, environmental protection, cooking, and health aspects of insect foods were higher compared with the perceptions of the low FD group. Furthermore, there were significant differences between the two groups across these dimensions. This was also reflected in the risk of consuming insect foods in the high and low FD groups.

Overall, among university students, the high FD group was more unwilling to consume insect foods than the low FD group. Both groups agreed on the benefits of insect foods but also believed that consuming insect foods had higher risks.

## 4 Discussion

In Asian countries, especially in parts of China, Thailand, and India, as well as in parts of Latin America and Africa, consumers have a custom of consuming insect foods based on tradition and dietary preferences. The consumption of edible insect foods is a sustainable alternative to traditional meat, and has reached a global consensus (28).

Experiencing disgust toward insect foods is a primary obstacle to the consumption of insect foods in Western countries (29). Disgust is an oral sensation or mental aversion caused by something dirty or disgusting. It manifests itself as distancing oneself from certain disgusting and potentially dangerous objects, events or situations, and is conceptualized as an adaptive response that can be changed based on information and life experiences (30). Consumers’ fear of unfamiliar food (FN) limits their likelihood of consuming insects (13). According to reports, the level of FN among Chinese college students is relatively high (31, 32), which is measured using the FN Scale (33). In this study, only 5.6% of college students were familiar with insect foods. Even in China, which has a tradition of consuming insect food, most Chinese college students were unfamiliar with insect food, which may be an important factor influencing college students’ unwillingness to consume. FN and the disgust or aversion toward or fear of insect foods has the strongest impact on respondents’ willingness and intention to consume insect foods (34). Furthermore, unfamiliarity with insect food exacerbates FN and FD toward insect food.

Therefore, to eliminate negative stereotypes about insect foods, positive cognitions surrounding insect foods must be maximized (35). Individual acceptance of new foods largely depends on their level of fear toward them. Consumers must be offered more opportunities to breed or contact with edible insects (36) and taste insect foods to improve their acceptability (37). Chefs can provide customers with a positive insect foods based cooking experience, thereby increasing their acceptance of insect foods (38). Such positive experiences can alleviate the negative perceptions of insect foods and cultivate positive attitudes toward insect foods. Furthermore, social media platforms should be used as potential tools for nutrition education on edible insect foods in the human diet to increase publicity efforts in favor of insect foods, transform personal ways of thinking, and make consumers believe that the insect foods made from edible insects are enticing and nutritious, instead of being considered ugly; this can maximize insect foods’ public awareness and acceptance (39). Consumers’ health literacy potentially influences their willingness to consume insect-based foods (40). Canadian high school students’ acceptance of edible insect foods increased substantially after participating in an educational insect-farming program (41).

Attention should be paid to innovations in the taste and cooking methods of insect-based foods. Through careful processing and packaging, insect foods can be portrayed as a high-end healthy food, substantially overcoming the original appearance of insect foods (42, 43). This can help reduce consumers’ fear and nausea of new foods and attract consumers who value the nutritional value of insect foods (44). Visual appearance plays an important role in influencing consumers’ acceptance of insects as food (45). Participants from France and Ireland prefer to consume delicious or disguised (invisible) insect foods rather than insects presented in their usual form (whole) (27). On 21 January 2025, according to the official announcement of the European Union, the European Commission issued Regulation (EU) No. 2025/89, approving UV-treated whole yellow mealworm powder as a new type of food to be put on the market for use in various food products, such as cheese, jam, and bread. This was in accordance with Regulation (EC) No 2015/2283 of the European Parliament and the Council.

Finally, ensuring the health and safety of insect-based foods is important. The risks of consuming insect foods are the same as those of consuming other protein sources, including biological risks (bacteria, viruses, and parasites), chemical risks (heavy metals, toxins, and pesticides), and allergens related to the environment (46). Heterologous proteins in insects are more likely to cause allergies than dairy and meat proteins, and some individuals may develop allergic reactions to insect-based foods. Insect viruses and bacterial risks can be controlled through modern processing. Currently, insect food regulatory agencies and related regulations are lacking worldwide. Therefore, it is necessary to improve the market supervision mechanisms to ensure the quality and safety of insect foods.

### 4.1 Limitations

This study had some limitations. First, the 560 university students in the sample were from one university in China, which could not provide an overview of the impact of FN and FD on insect food consumption among Chinese university students. Second, the online self-report questionnaire may have led to some bias in the survey results. However, the heterogeneity of university students with different majors and places of residences still provide insights into their insect food consumption. Future research should focus on the following aspects. First, the main communication factors (e.g., social media publicity, peer influence, internet communication, or a combination of these) of Chinese university students’ insect FD or FN should be examined. Second, cutting-edge technologies, such as artificial intelligence and wearable devices, should be adopted to study the intensity and dynamic changes in college students’ fear or disgust surrounding different insect foods as well as their impact on psychological emotions. This can help break through the singularity and bias of self-assessment questionnaires (47, 48).

## 5 Conclusion

This study aimed to evaluate the impact of FN and FD on university students’ willingness, benefits, and risks of insect food consumption. Overall, most university students were unfamiliar with insect foods and unwilling to consume them, especially those with high levels of FN and FD. Regardless of the FN and FD levels, both the high and low groups agreed on the benefits of consuming insect foods but had higher risk perceptions of consuming insect foods. In the future, the government, universities, colleges, and communities will have to promote and enhance the positive cognitions surrounding insect foods among university and college students and their parents through lectures, practical experiences, and social media. They will also be required to eliminate negative stereotypes about insect foods, adopt scientific and technological innovation to ensure the health and safety of insect foods, minimize the consumption risks of insect foods, and increase students’ willingness to consume insect foods, especially those with high FN or FD.

## Data Availability

The original contributions presented in this study are included in this article/Supplementary material, further inquiries can be directed to the corresponding author.
