# Supplementary material for: Association of food neophobia and food disgust with the willingness, benefits, and risks of insect food consumption among Chinese university students
Source: Front Nutr. 2025 Jul 14;12:1613932. doi: 10.3389/fnut.2025.1613932 (PMC12319225; doi:10.3389/fnut.2025.1613932)
Supplement: Supplementary file 1 [file Data_Sheet_1.docx]

**Supplementary materials**

Supplementary Table 1 Food neophobia scale

| Items | |
| --- | --- |
| FNS1 | I am constantly sampling new and different foods (R) |
| FNS 2 | I don’t trust new foods |
| FNS 3 | If I don’t know what a food is, I won’t try it |
| FNS 4 | I like foods from different cultures (R) |
| FNS 5 | Ethnic food looks weird to eat |
| FNS 6 | At dinner parties, I will try new foods (R) |
| FNS 7 | I am afraid to eat things I have never had before |
| FNS 8 | I am very particular about the foods I eat |
| FNS 9 | I will eat almost anything (R) |
| FNS 10 | I like to try ethnic restaurants (R) |
| 5-point Likert scale: | |
| - Ranging from 1 for “strongly disagree” to 5 for “strongly agree.” - Each item score varies from 1 to 5. - The total score varies from 10 to 50. - A higher score indicates a higher food neophobia level. - ‘R’ stands for ‘reverse item’. | |

Supplementary Table 2 Food disgust scale

| Items | | |  |  |
| --- | --- | --- | --- | --- |
| FDS 1 | Uncooked animal bones in the mouth | |  |  |
| FDS 2 | Eating with dirty dishes in a restaurant | |  |  |
| FDS 3 | Food donated by an unfamiliar neighbor | |  |  |
| FDS 4 | Eating hard cheese with mold removed | |  |  |
| FDS 5 | Eating apple slices that turned brown when exposed to air | |  |  |
| FDS 6 | Eating unwashed live fish | |  |  |
| FDS 7 | Eating discolored avocado flesh | |  |  |
| FDS 8 | Eating a salad with a snail | |  |  |
| 5-point Likert scale:   - Ranging from 1 for “very not disgusted” to 5 for “strongly disgusted”. - Each item score varies from 1 to 5. - The total score varies from 8 to 40. - A higher score indicates a higher food disgust level. | | |  | |

Supplementary Table 3 University students’ willingness, benefits, and risks of insect food consumption

| Items | |
| --- | --- |
| Willingness | |
| W1 | I won’t eat at any time. |
| W2 | I will only eat if I have to rely on it for survival. |
| W3 | I’m not sure if I will consume it. |
| W4 | Can be persuaded to consume. |
| W5 | I am happy to consume. |
| Benefits |  |
| Nutrition | High protein content, low fat content. |
| Rich or available | Abundant sources. |
| Cost | Breeding costs are lower than raising cattle. |
| Environmental protection | Reduced the impact of the commercial meat industry on the environment. |
| Cooking | Increased consumer choices. |
| Health | Possible health benefits. |
| Risks |  |
| Microorganisms or diseases | May be infected with diseases or transmitted by insects. |
| Food Fear | Insects are disgusting. |
| Poison or toxin | May cause food poisoning. |
| Sensory dislike | Taste may not be acceptable or texture may not be good. |
| Insecticides or chemicals | Residual chemicals and insecticides on the body. |
| Allergy | May cause body allergies. |
| General health risks | Can affect our health. |
| 5-point Likert scale:   - Ranging from 1 for “strongly disagree” to 5 for “strongly agree”. - Each item score varies from 1 to 5. | |

Supplementary Table 4 Familiarity with and selection of insect food.

| Items | | Answers |
| --- | --- | --- |
| 1 | Are you familiar with insect food? | There are five answers to choose from, as follows 1=very unfamiliar, 2=unfamiliar, 3=neutral, 4=familiar, 5=very familiar. |
| 2 | Please choose insect food that you frequently consume? | There are ten answers to choose from, as follows ants, tenebrio molitor, cicada and silkworm pupae, clanis bilineata, grasshopper, cricket, maggots, scorpion, other, never eat. |
| 3 | Possible reasons for disliking eating insect food. | There are five answers to choose from, as follows unfamiliar, bad taste, disgusting, health risks, unethical. |
